# Biodegradable Films of PLA/PPC and Curcumin as Packaging Materials and Smart Indicators of Food Spoilage

**DOI:** 10.1021/acsami.2c02181

**Published:** 2022-03-18

**Authors:** Martin Cvek, Uttam C. Paul, Jasim Zia, Giorgio Mancini, Vladimir Sedlarik, Athanassia Athanassiou

**Affiliations:** † Centre of Polymer Systems, University Institute, Tomas Bata University in Zlin, Trida T. Bati 5678, 760 01 Zlin, Czech Republic; ‡ Smart Materials, Istituto Italiano di Tecnologia, Via Morego 30, 161 63 Genoa, Italy

**Keywords:** polylactic acid, poly(propylene carbonate), curcumin, smart food packaging, chemical sensor, bioplastics, indicator

## Abstract

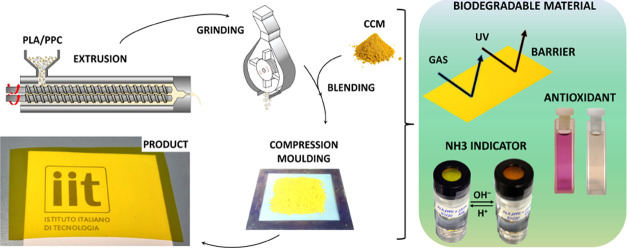

Bio-based and biodegradable packaging combined with chemical sensors and indicators has attracted great attention as they can provide protection combined with information on the actual freshness of foodstuffs. In this study, we present an effective, biodegradable, mostly bio-sourced material ideal for sustainable packaging that can also be used as a smart indicator of ammonia (NH_3_) vapor and food spoilage. The developed material comprises a blend of poly(lactic acid) (PLA) and poly(propylene carbonate) (PPC) loaded with curcumin (CCM), which is fabricated via the scalable techniques of melt extrusion and compression molding. Due to the structural similarity of PLA and PPC, they exhibited good compatibility and formed hydrogen bonds within their blends, as proven by Fourier transform infrared (FTIR) and X-ray diffraction (XRD). Thermogravimetric analysis (TGA) and differential scanning calorimetry (DSC) analysis confirmed that the blends were thermally stable at the used processing temperature (180 °C) with minimal crystallinity. The rheological and mechanical properties of the PLA/PPC blends were easily tuned by changing the ratio of the biopolymers. Supplementing the PLA/PCC samples with CCM resulted in efficient absorption of UV radiation, yet the transparency of the films was preserved (*T*_700_ ∼ 68–84%). The investigation of CCM extract in ethanol with the DPPH^•^ assay demonstrated that the samples could also provide effective antioxidant action, due to the tunable release of the CCM. Analyses for water vapor and oxygen permeability showed that the PPC improved the barrier properties of the PLA/PPC blends, while the presence of CCM did not hinder barrier performance. The capacity for real-time detection of NH_3_ vapor was quantified using the CIELab color space analysis. A change in color of the sample from a yellowish shade to red was observed by the naked eye. Finally, a film of PLA/PPC/CCM was successfully applied as a sticker indicator to monitor the spoilage of shrimps over time, demonstrating an evident color change from yellow to light orange, particularly for the PPC-containing blend. The developed system, therefore, has the potential to serve as a cost-effective, easy-to-use, nondestructive, smart indicator for food packaging, as well as a means for NH_3_ gas monitoring in industrial and environmental applications.

## Introduction

In food packaging, the current production trends, associated with consumer demands and food waste reduction, have led to the development of “smart” packaging products. Such systems are capable of providing conventional passive functionality, physically protecting foodstuffs from unwanted external factors, but also interacting with the food environment in a controllable manner.^1,2^ Interaction usually occurs between a sensing element in the packaging and foodborne chemicals, inducing visible changes in the color of the former, thereby providing information about the real-time freshness of the product.^3^

All types of foodstuffs, especially products with fish and meat, generate a wide range of chemicals during spoilage. This stems from the complexity of the spoilage process that encompasses chemical and biological alteration of protein and lipid profiles.^4^ As a consequence, analyzing food quality requires a special laboratory and experienced technicians, in addition to being destructive and time-consuming.^5^ Alternative methods, such as the electronic nose, necessitate specific treatment of samples prior to analysis.^5,6^ In light of such demanding requirements, there has been a rise in the utilization of smart indicators that are sensitive, nondestructive, compact, inexpensive, easy to use, and easily scalable for industrial production.^3^ In this respect, bio-based, sustainable, and biocompatible indicators that enable detection by the naked eye stand out as promising systems for smart, food packaging indicators.

A chemical indicator or sensor is usually composed of a sensitive pigment/dye (the active component) and a solid support.^7^ The active components can be immobilized onto the solid part by physical adsorption, attached covalently, or physically entrapped inside the polymeric matrix of the support.^8^ Natural pigments are always preferred over their synthetic analogues (e.g., bromophenol blue, bromocresol purple, chlorophenol red), as they do not pose any threat to human health.^3,9^ Indeed, a great deal of effort has been devoted to the study of anthocyanins, which are frequently applied in the colorimetric determination of pH variations in food products.

Anthocyanins are phenolic compounds that naturally occur in certain plants.^7^ For instance, anthocyanins extracted from red cabbage (*Brassica oleraceae*) were integrated into bacterial cellulose membranes and tested across a wide range of pH.^7^ They were also applied in a detector for fish spoilage based on chitosan/corn starch.^10^ Choi et al.^9^ investigated extracts from purple sweet potato (*Ipomoea batatas*) embedded in an agar/potato starch matrix to discern the freshness of pork. An analogous system was developed to detect pork freshness, utilizing anthocyanins from roselle (*Hibiscus sabdariffa*) and films of starch/poly(vinyl alcohol) (PVA)/chitosan.^11^ The jambolan plum (*Syzygium cumini*) was recently employed as a source of anthocyanins, which after being embedded in a chitosan/PVA blend was capable of monitoring the freshness of shrimps.^12^ Although anthocyanins have been promising for the development of such indicators, their limited stability when exposed to relatively high temperatures (even around 60 °C) and other external factors (e.g., light, oxygen)^13^ not only restricts their applicability but also could negatively affect the reliability of the sensing elements produced.

Curcumin (CCM), 1,7-bis (4-hydroxy-3-methoxyphenyl)-1,6-heptadien-3,5-dione, is a natural polyphenolic phytochemical that is extracted from herbaceous turmeric and curcuma rhizomes. This compound has been used in a variety of applications as it exhibits antioxidant, antimicrobial, antiviral, anti-inflammatory, and other important physicochemical and pharmacological activities.^14−16^ CCM is also highly responsive to pH variations demonstrating remarkable colorimetric changes.^17^ For this reason, it has been successfully adopted as a pH- and NH_3_-indicating substance in κ-carrageenan-based^14^ and tara gum/PVA-based^17^ films, applicable in the food industry. The composites were however produced by laboratory methodologies, such as solution casting, which are not usually applicable at an industrial scale. Also, other bio-active formulations with CCM have been fabricated via casting methods^18−20^ or swell-encapsulation-shrink techniques.^21^ Initial attempts to use industrially applicable methods for the fabrication of composites containing CCM were recently made by Zia et al.,^22^ who applied melt extrusion at 145 °C for composites of low-density polyethylene (LDPE) without degrading the CCM. The current trend in food packaging however requires the utilization of biodegradable biopolymers since their conventional petroleum-based counterparts have caused serious environmental issues due to inappropriate waste management.^23^ For the aforementioned reasons, there is a significant demand to develop environmentally friendly, colorimetric indicators, applicable in smart food packaging industry that can be produced by large-scale techniques.

In this paper, we report on the development of films consisting of blends of poly(lactic acid) (PLA)/poly(propylene carbonate) (PPC) loaded with small amounts of CCM, intended for the smart packaging of foodstuffs. PLA is a synthetic biopolymer that has received tremendous attention for its excellent processability, high Young modulus, good optical properties, industrial compostability, and renewable origin.^24,25^ Despite these positive aspects, PLA holds some shortcomings from which, limited ductility is probably the most striking one.^26^ As a consequence, plasticization with different low-molecular compounds^27^ or other ductile biodegradable polymers is necessary to produce flexible films. PPC is a biodegradable polymer derived from CO_2_ and propylene oxide, and its ester units exhibit high chain flexibility and good flow properties.^28^ Recently, the PLA/PPC blends were prepared by casting method using chloroform, a hazardous solvent.^29−31^ Although this technique is convenient for laboratory-scale prototyping with low production volumes, it is not widely applicable at the industrial level.^32^ Moreover, the use of chloroform in food packages, drugs, and cosmetics is strictly banned by The Food and Drug Administration because of evidence indicating it may cause cancer.^33^ To address these limitations, we adopted melt extrusion and compression molding as an effective “green” strategy to obtain the homogeneous PLA/PPC blends with the potential for upscale production.

The physicochemical behavior of the proposed packaging films and indicators was studied in detail. Their mechanical properties were tuned by simply varying the PLA/PPC ratio. The immobilized CCM allowed the capability of the samples to sense NH_3_ vapors and food spoilage, while still preserving high optical transparency. Furthermore, the CCM molecules acted as a UV-light barrier, potent antioxidant, and radical scavenging agent, while the CCM did not impair the barrier properties of the biodegradable films. An experiment was conducted with the developed indicator to monitor the freshness of shrimps, showing potentiality for real-time monitoring food spoilage. The films fabricated herein could be used as complete packaging, but could also be easily integrated into the lid of a food container, as a result of their compactness and easy-to-use features.

## Experimental Section

### Materials

Thermoplastic PLA 2003D was purchased from NatureWorks LLC (Minnetonka). The molecular weight, *M*_w_, of the neat PLA pellets ranged between 87–97 kDa, with a polydispersity index, *Đ*, of about 1.40–1.54 and specific gravity of 1.24 g/cm^3^. The PPC was purchased from Empower Materials (QPAC 40), had *M*_w_ of 100–300 kDa and specific gravity of 1.26 g/cm^3^. Both polymers were dried at 40 °C for 24 h prior to processing. Curcumin (from Curcuma longa, turmeric powder), hydrochloric acid (HCl; reagent grade, 37%), sodium hydroxide (NaOH; BioXtra, ≥98.0%, pellets, anhydrous), ammonium hydroxide solution (33–35% NH_3_ in H_2_O), poly(2,6-diphenyl-*p*-phenylene oxide), i.e., Tenax; (particle size: 60–80 mesh, surface area: ∼35 m^2^/g), ethanol (96%) and 2,2-diphenyl-1-picrylhydrazyl (DPPH; 95%) were obtained from Sigma (Sigma-Aldrich) and utilized as received. Fresh shrimps were purchased from a shop in the Liguria region of Italy. Ultrapure water from Milli-Q (Advantage A10, Millipore) was applied throughout the study.

### Fabrication of the Films

The neat PLA and PLA/PPC blends with 20 and 40 wt % of PPC were processed in a twin-screw Rheoscam extruder (Scamex, France), with a screw diameter of 20 mm and a length-to-diameter (L/D) ratio of 20. The heating profile from the feed section to the extrusion die comprised a gradually increasing temperature from 160 to 180 °C (temperature profile was set to 160, 170, 170, 170, 175, and 180 °C, respectively), which was identical for all of the blends, and the rotation speed was constantly 75 rpm. The temperature of the feed throat was maintained at 45 °C by means of a Ministat 125 thermostat unit (Huber, Germany). Filaments were produced by extrusion through a circular dye of 2 mm in diameter, pelletized, and thoroughly mixed with the CCM powder (2 wt %). Next, the blends were compression molded in a Carver press (Model 3853CE) with a hydraulic pump. The blends were compressed with a pressure of 10 MPa, for 5 min, at 175 °C. The samples returned to laboratory temperature in a controlled manner to ensure the repeatability of the process. The thickness of the produced films was 0.51 ± 0.03 mm.

### Fourier Transform Infrared (FTIR) Spectroscopy

FTIR was conducted on a Vertex 70v vacuum spectrometer (Bruker), equipped with the attenuated total reflectance (ATR) accessory and diamond crystal. Spectra were recorded in the wavenumber region of 4000 to 600 cm^–1^ across 64 scans and at the spectral resolution of 2 cm^–1^. The curvature of each spectrum was treated using the baseline correction (4 points for a new baseline definition). The corrected spectrum was normalized and smoothed. All data were processed in OPUS software.

### Scanning Electron Microscopy (SEM)

The cross-sectional microstructure of the samples was investigated on a Nova NanoSEM 450 field emission scanning electron microscope (FEI, Japan) equipped with an ETD detector, set to an accelerating voltage of 5 kV. Prior to analysis, the samples were freeze fractured using liquid nitrogen (N_2_) and placed onto an aluminum pin stub. A thin layer of gold (10 nm) was deposited on the surfaces of the samples by an SC7620 sputtering device (Quorum Technologies, UK) to avoid the surface charging effect.

### X-ray Diffraction (XRD)

The crystallography of the samples was examined via XRD analysis using Miniflex 600 (Rigaku, Japan) diffractometer with a Co-Kα radiation source (λ = 1.789 Å) operating within the 2θ range of 10–60° with a scan speed of 3°/min.

### Thermogravimetric Analysis (TGA)

TGA was carried out on a TGA Q500 device (TA Instruments) to determine the thermal stability of the CCM and PLA/PPC blends. A small amount (5–15 mg) of each sample was placed into platinum pans and subjected to a range of temperature from 30 to 800 °C, at a heating rate of 10 °C/min. The chamber was purged with N_2_ at a flow rate of 50 mL/min. The mass loss of each sample was recorded as a function of temperature.

### Differential Scanning Calorimetry (DSC)

Thermal phase transitions were investigated by DSC on a Diamond-DSC unit (PerkinElmer). Prior to taking measurements, the instrument was calibrated using the Indium standard with a melting point of 156.6 °C. The samples (5–10 mg) were sealed in aluminum pans and subjected to a defined temperature ramp, from −20 to 200 °C, with a heating/cooling rate of 10 °C/min, under a nitrogen purge of 20 mL/min. Isothermal steps of 1 min were employed to equilibrate the samples at the interval boundary temperatures. The thermal history of the samples was quenched during the first heating cycle, while data from the second heating scan were used to determine the glass-transition temperature (*T*_g_), cold crystallization temperature (*T*_cc_), enthalpy of cold crystallization (Δ*H*_cc_), melting temperature (*T*_m_) and enthalpy of melting (Δ*H*_m_). The degree of crystallinity (χ_C_) was calculated according to the following formula
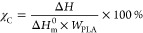
1where Δ*H* equals (Δ*H*_m_–Δ*H*_cc_), Δ*H*_m_^0^ is the melting enthalpy of the 100% crystalline PLA (93.0 J/g), and *W*_PLA_ is the weight fraction of the PLA.^34^ The calculation was made in consideration of evidence that PPC was an amorphous polymer.^35^

### Melt Rheology

The flow properties of the molten-state samples were investigated on a modular rheometer (Physica MCR502, Anton Paar, Austria) coupled with a CTD600 heating chamber and TC 30 temperature control unit. The experiments were conducted using the parallel-plate geometry of 25 mm in diameter and a gap of 0.35 mm. The viscoelastic data were collected over an angular frequency sweep from 0.3 to 300 rad/s at a strain amplitude of 0.02% and 180 °C (the processing temperature). The linearity of the response was verified. Nitrogen was directed into the measuring chamber (shaft 200 L_N_/h, cooling of 1.0 m_N_^3^/h) to limit the thermo-oxidation of the samples when acquiring the measurements. An empirical Cole–Cole model for complex dynamic viscosity, η*, was applied to enable the analysis of the data.^36^ The mathematical form of the Cole–Cole equation is expressed as
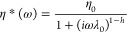
2where η_0_ is zero-shear viscosity, ω is the angular frequency, λ_0_ is the mean relaxation time, *h* is the parameter reflecting the relaxation time distribution, and *i* is the imaginary number (*i*^2^ = −1).

### Mechanical Properties

The tensile properties of the PLA/PPC blends and their CCM-loaded analogues were examined on an Instron 3365 device (Instron), equipped with a 5 kN load cell at a crosshead speed of 5 mm/min. Specimens in the form of tensile bars (length 35 mm, width 4 mm) were cut out from the films and tested according to ASTM D882-12. One-way analysis of variance (ANOVA) and *t*-test were used to statistically evaluate the significance of the data at a 5% significance level. The results were reported as mean values and standard deviations from six measurements.

### Water Contact Angle (WCA)

WCA measurements of the PLA/PPC films and their analogues with CCM were recorded on an optical, high-speed, contact angle measuring system (OCAH-200, Dataphysics, Germany) via the static sessile drop method. The instrument was equipped with a vertically adjustable table, a blunt needle (inner diameter of 0.52 mm), and an electronic dosing system, the latter facilitating accurate droplet (5 μL) deposition on the substrate. Images were captured on a CCD camera (640 × 480 pix) and evaluated by built-in software (SCA 20, version 2). Each sample was tested 10 times in random locations, with results expressed as the mean value and standard deviation.

### Water Vapor Permeability

The water vapor transmission rate (WVTR) and water vapor permeability (WVP) of the PLA/PPC films and their analogues with CCM were determined gravimetrically according to the ASTM E96 standard test method.^37^ The investigation was carried out at ambient temperature, under 100% relative humidity gradient (ΔRH%). Deionized water (400 μL) (which generates 100% RH inside a permeation test cell) was added into each such cell of given dimensions (internal diameter of 7 mm, inner depth of 10 mm). The films were cut into circles with a cutting press and put on top of the permeation test cells. The cells were placed inside a desiccator containing anhydrous silica gel, which served as a desiccant for maintaining 0% RH.^37,38^ The water vapor transferred through the film and absorbed by the desiccant was determined from the change in weight of the cells at hourly intervals over an 8 h period. A set of electronic scales (accuracy of ±0.1 mg) was employed to record loss in weight over time, and these values were plotted as a function of time. The WVTR was determined from the slope of each line obtained from linear regression according to the following equation
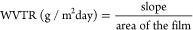
3

WVP measurements were replicated three times for each PLA/PPC film and their composites with CCM. Values for WVP were calculated by the following equation^37−39^
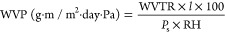
4where *l* (m) is the mean film thickness measured with a micrometer (accuracy of 0.001 mm), ΔRH (%) is the relative humidity gradient percentage, and *P*_S_ (Pa) denotes WVP saturation at 25 °C.

### Oxygen Permeability

The oxygen permeation of the PLA/PPC films and their CCM-loaded analogues was tested on an Oxysense 5250i device (Oxysense), equipped with a film permeation chamber. The instrument was operated according to Method-F 3136–15 (ASTM, 1989). Testing took place under standard laboratory conditions, i.e., 21 ± 2 °C and 50 ± 2% RH. The permeation chamber comprised a cylinder divided into 2 parts, i.e., wells for sensing and driving. The films were cut into a rectangular shape (6 cm × 6 cm), placed over the sensing well, and the chamber properly sealed by the locking bolts. The instrumentation of the well included a fluorescence sensor (oxydot) mounted to the side of the chamber purged with nitrogen, whereas the driving well is kept open to ambient air. The OxySense fiber-optic pen measures the oxygen reading from the oxydot, at specific time intervals. The oxygen volumetric flow rate per unit area of the film and time unit (oxygen transmission rate (OTR), mL m^–2^ day^–1^) was continuously monitored until the steady-state was achieved. The OxySense software translated these data to determine the OTR of the films. At least ten readings for each sample were recorded with a minimum correlation coefficient (*R*^2^) value of 0.995. The oxygen permeability (OP) of the films was then calculated according to the following equation
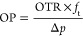
5where OTR is the oxygen transmission rate, *f*_t_ is the thickness of the film, and Δ*p* is the oxygen partial pressure difference between the sides of the films.

### Optical Properties

UV–vis spectroscopy was performed to discern the optical properties of the PLA/PPC films and their CCM-loaded analogues. The thickness of the samples was decreased to 50 μm to match that of common packaging films, such as polyolefins.^40^ Spectra were collected on a Cary 6000i UV–vis spectrophotometer (Varian) operating in transmission mode, within the spectral range of 200–800 nm at the resolution of 2 nm.

### Release Kinetics for the Active Indicators

UV–vis spectroscopy was applied to determine the kinetics of release for CCM from the PLA/PPC indicator films and antioxidant activity, in accordance with Zia et al.^22^ To assess the release kinetics, the PLA/PPC indicator films containing CCM were placed in an ethanol solution (96%), at the concentration of 1% (w/v), and change in absorbance of the extracted CCM present in the solution was recorded after predetermined intervals for a period of 24 h. The absorption of the solutions was recorded on a Cary 6000i spectrophotometer with identical parameters to the previous test (see the Optical Properties section). Pure ethanol was utilized for baseline correction during the experiments. The mean value for absorbance at 428 nm (the characteristic peak for CCM) for three different samples was reported for each time interval.^22^ The concentration of the released CCM in the solution was evaluated by an equation gained after constructing a linear fit (Figure S1) from data gathered on the absorption spectra of five different CCM solutions with known concentrations of CCM ranging between 1–5 μg/mL. According to the linear fit, the concentration of CCM in the unknown solutions was calculated by the following equation:
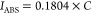
6where *I*_ABS_ is the intensity of the absorbance peak at 428 nm and *C* is the CCM concentration.

### Antioxidant Activity of the Films

The antioxidant activity of the PLA/PPC films supplemented with CCM was determined by the DPPH^•^ free radical scavenging method.^22^ First, CCM extract was collected from various vials containing the film samples (1% (w/v)) at predefined intervals during 24 h. Then, 2 mL of the CCM extract solution was mixed with 2 mL of the DPPH^•^ assay (50 μM solutions) in ethanol and left in the dark for 30 min; the absorbance of this solution was subsequently investigated. The absorbance of the second solution was measured by mixing 2 mL of the CCM extract solution with 2 mL of ethanol. Finally, the spectrum for absorbance was obtained by mixing 2 mL of the DPPH^•^ assay (50 μM solutions) in ethanol with 2 mL of ethanol. The antioxidant activity was calculated according to the following equation
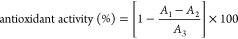
7where A_1_ is the absorbance value at 517 nm of the solution with the CCM extract and DPPH^•^ assay, A_2_ is the absorbance at 517 nm of the CCM extract with ethanol, and *A*_3_ refers to the absorbance of the DPPH^•^ control solution at the same wavelength. All results are reported as the mean values for different samples in triplicate.

### Migration Analysis

As for other food contact materials,^41^ migration analysis from the PLA/PPC blends and their analogues with CCM was determined using Tenax as a simulant of dry food, in accordance with Commission Regulation (EU) 10/2011. The methodology for analysis was adapted from our previous works.^42,43^ Circular samples of the sensor films, 16 mm in diameter, were put into clean glass Petri dishes, and 40 mg of Tenax was placed on each side. The vial was sealed and exposed to 40 °C for 10 days in a vacuum oven. Finally, the samples were removed and cooled to laboratory temperature. Values for overall migration (OM) were obtained by calculating the mass difference of Tenax before and after treatment, applying the following formula
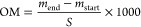
8where *m*_start_ and *m*_end_ represent the weight of Tenax at the beginning and end of the test; *S* denotes the surface area of the test specimen intended to come into contact with the given foodstuff, in dm^2^; and accuracy was ensured by performing the test in triplicates.

### Detection of Ammonia

A test for detecting ammonia (NH_3_) vapors was performed to examine the efficiency of the PLA/PPC chemical indicators loaded with CCM during a process of simulated spoilage. Circular-shaped samples with a diameter of 16 mm and thickness of 0.3 mm were cut out from the sheets and integrated within the stoppers of vials that contained aqueous ammonia solution at 10 vol %. The experiment was performed under laboratory conditions. Each assembly was fitted with a rubber seal to prevent the leakage of NH_3_ vapor from inside.

### CIELab Color Analysis of PLA/PPC Indicators

CIELab color space is an analytical tool that mimics the human eye color perception.^44^ In this study, CIELab space was employed as a scientific proof of the color changes produced by the PLA/PPC indicator films. The CIELab color space consists of three color components known as tristimulus values, represented by lightness of the color (L*) and its hue (a* and b*). The values of L*, a*, and b* were collected from the images taken using a reflex camera (Canon EOS 5D Mark II, Japan) under the same light and distance using the free mobile application “deltacolor”. The total color difference (Δ*E*) was calculated from the following equation^45^
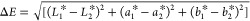
9

When the value of Δ*E* is greater than 3.5, the color difference is clearly perceivable by the experienced and unexperienced observers.^45,46^

### Food Spoilage Test

A test of food spoilage was performed to verify the potential of the CCM-loaded PLA/PPC indicators for practical application. The indicators were attached to the covers of a Petri dish with double-sided adhesive tape. Fresh shrimps (100 g) were put in the dish, which was sealed with Parafilm and left for 5 days at the laboratory conditions. The change in color of the indicators was inspected after this period had elapsed using the CIELab color space analysis, similarly to the above.

## Results and Discussion

### Design and Development of Biodegradable Films

**Figure 1:**
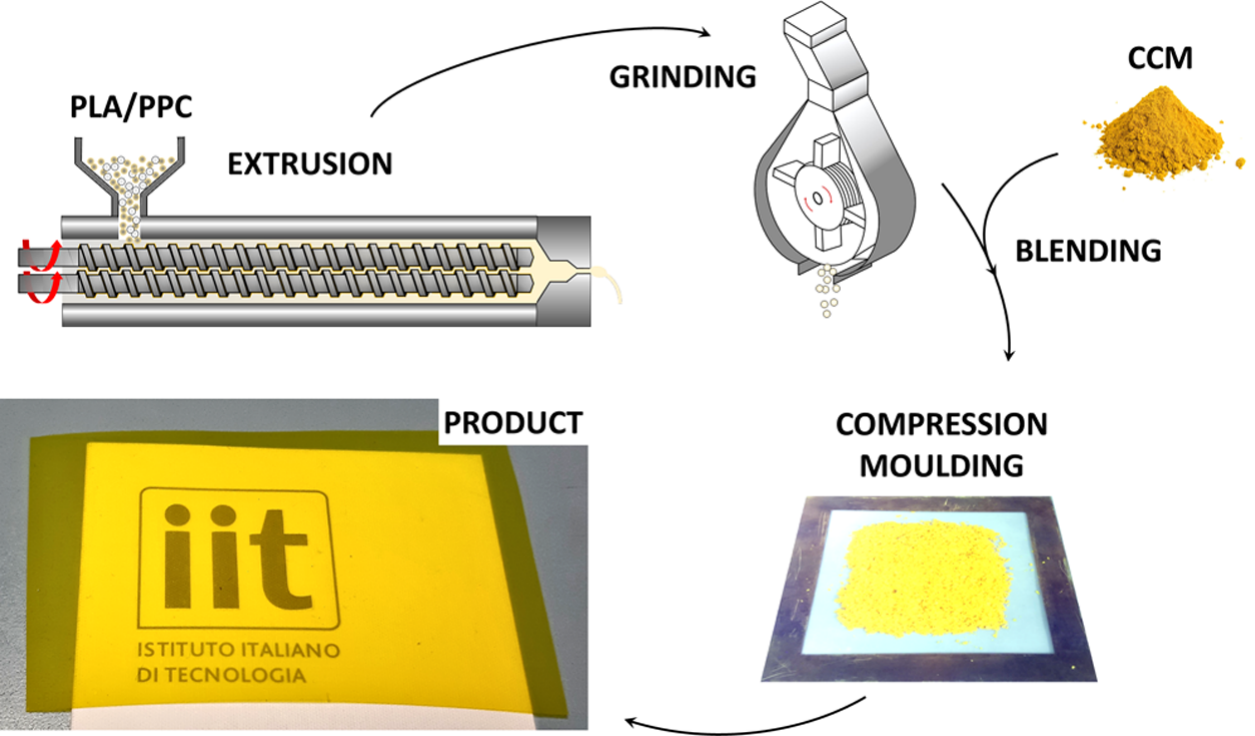
Schematic representation of the fabrication process, comprising methodologies suitable for large-scale production. Macroscopic views of the CCM-loaded PLA/PPC pellets and the final product.

The CCM component was mechanically mixed thoroughly with the extruded PLA/PCC pellets that were ground into a fine powder to minimize the time of its exposure to high temperatures and to preserve its activity (Figure 1). The CCM concentration of 2 wt % was selected, after preliminary tests, as the best compromise for appropriate mechanical, optical, and sensing performances of the indicators. When viewed by the naked eye, the samples appeared remarkably homogeneous with good dispersion of the CCM, which provided them with the typical yellow-orange hue, shown in Figure 1. The physicochemical properties of the developed samples were analyzed, with emphasis on properties critical for food packaging applications.

### Chemical Analysis

**Figure 2:**
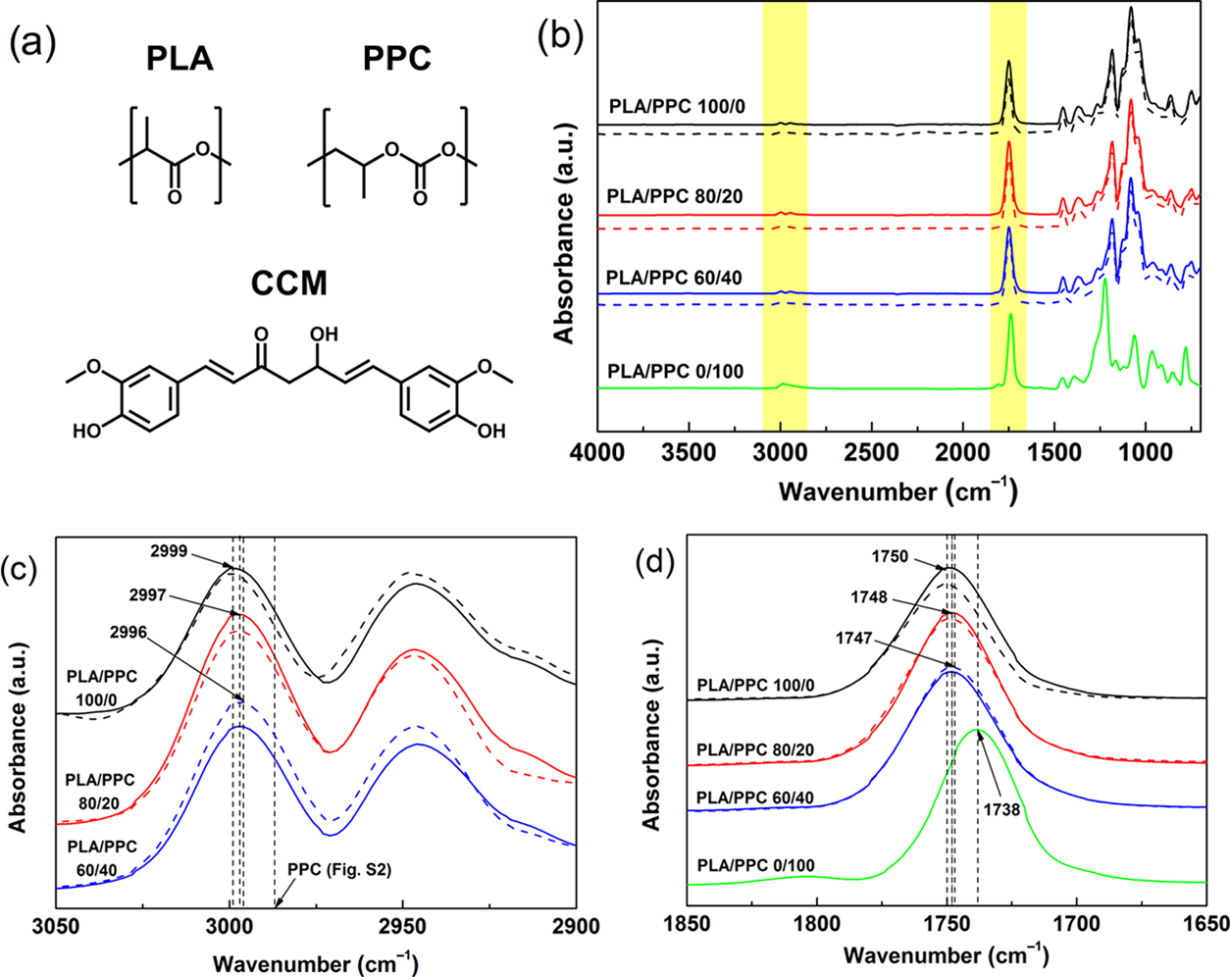
(a) Chemical structure of the PLA, PPC, and CCM molecules. (b) FTIR spectra of the PLA/PPC blends (solid lines) and their analogues with CCM (dashed lines). (c, d) Magnified FTIR spectra at the wavenumbers areas of interest, highlighted with yellow in (b).

The chemical compatibility of the components in the developed samples was examined by FTIR-ATR. Spectra for neat PPC and PLA were collected as references to identify any differences (Figure S2). Since the two polymers have a similar structure (Figure 2a), only slight variations between them were expected in their spectra (Figure 2b). Typical hydroxyl absorption in the region of 3700–3100 cm^–1^ was negligible due to a scarcity of terminal O–H groups.^47,48^ The peak corresponding to C–H stretching vibration at 2999 cm^–1^ for the PLA polymer (Figure 2c), shifted 3 cm^–1^ toward lower wavenumbers after the content of PPC was increased to 40 wt %. Such behavior is a consequence of intermolecular interactions between C–H and O–C–, or C–H and O=C– groups.^47^ A similar alteration was identified for the stretching of C=O groups (Figure 2d), where the related PLA peak at 1750 cm^–1^ shifted after the co-blending with PPC. This transition is explained by the aforementioned interaction between the groups of C–H and O=C–, C=O and C=O, or C=O and O–C.^47^ Further evidence of the PLA/PPC partial miscibility was recently demonstrated by Haneef et al.^29^ who investigated the entire range of blending ratios; our findings correlate well with the reported results. The presence of the CCM (dashed lines in Figure 2) slightly contributed to a shift of carbonyl stretching vibration of PLA, at 1750 cm^–1^, which was attributed to weak hydrogen bonding between the C=O groups in the polymer and OH groups in the CCM.^49^ Interaction with the CCM weakened after increasing the content of PPC, the shift was less evident, probably because of the reduced relative number of C=O groups in the PLA/PPC blend (per unit volume). FTIR analysis revealed some specific interactions between the PLA and PPC, indicating the chemical compatibility of their blends. The compatibility of the blends was also confirmed by complementary methods, discussed below.

### Microstructural Analysis

**Figure 3:**
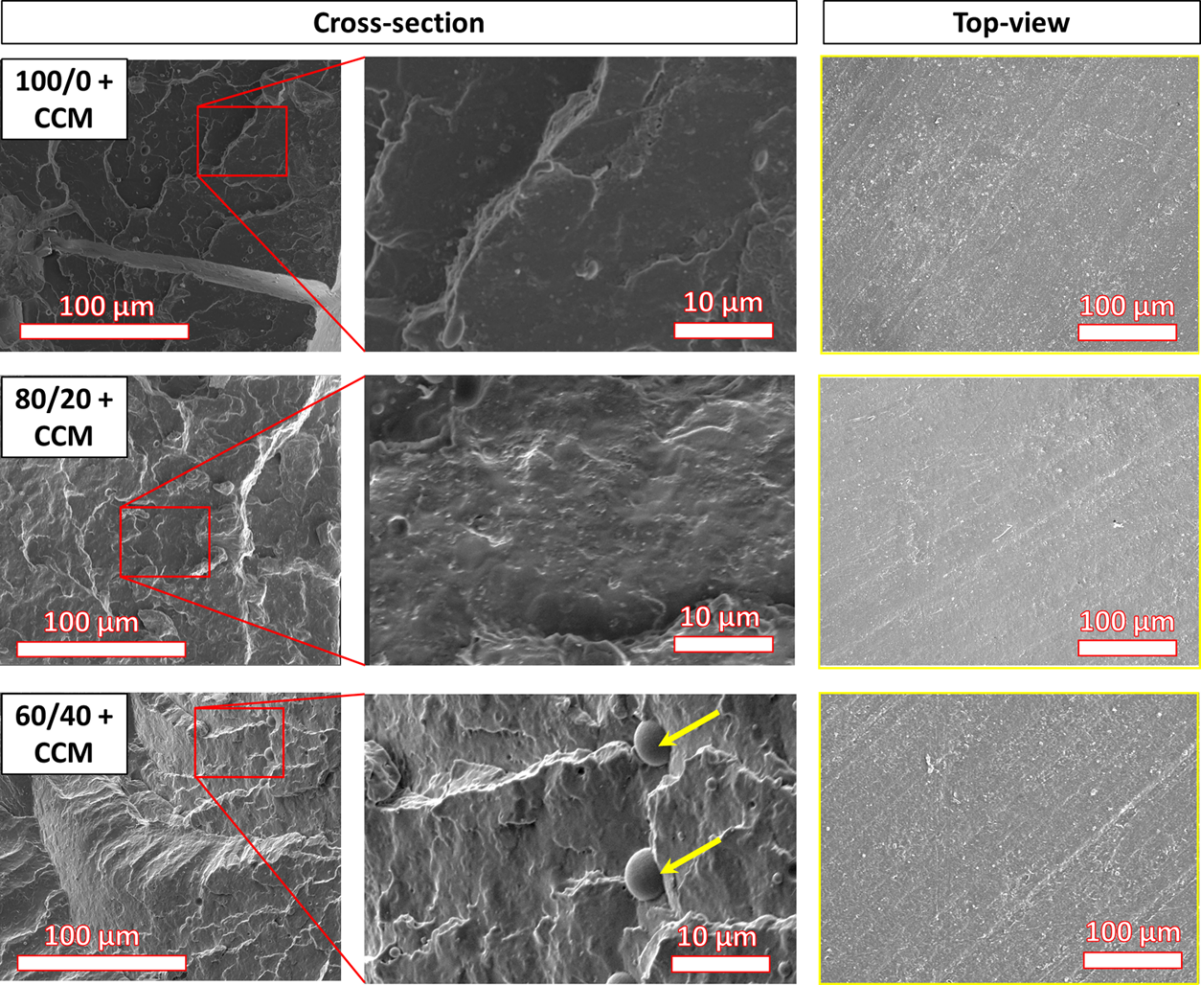
SEM micrographs of cross sections with magnified images, and of the surfaces, of PLA/PPC films loaded with CCM. The micro-phase separation of PPC in the sample containing 40 wt % PPC is denoted by the arrows.

The microstructures of the CCM-loaded PLA/PPC films were examined to evaluate both the compactness of the matrix and the level of dispersion of CCM. Figure 3 shows SEM micrographs of cryogenically fractured cross-sectional planes and of the surface of the samples. The PPC-containing films exhibited a somewhat rougher cross section than the neat PLA films. At greater magnification, the sample with 40 wt % PPC showed some micro-phase separation of the PPC into small clusters within the PLA matrix, in accordance with the literature.^47^ The SEM images of CCM-loaded samples were almost identical to those of their neat analogues (Figure S3), even though the powder CCM shows defined crystals with dimensions of 10–30 μm (Figure S4). Therefore, in PLA/PPC blends, CCM did not contribute with additional features and no aggregates were identified, suggesting it was evenly distributed throughout the matrix. CCM demonstrated compatibility with the matrix, as well as potentially good affinity and intermolecular binding, in good agreement with the FTIR data (Figure 2). Moreover, the XRD spectra of CCM-loaded PLA/PPC blends were absent of peaks coming from the CCM crystalline phase (Figure S5). A wide diffraction pattern from 10 to 30° was attributed to the scattering caused by the amorphous matrix.^50^ These findings confirm the compatibility of CCM with the blend matrix, at least at the concentration of 2 wt % applied herein. Of note is that Liu et al.^14^ described the formation of micron-sized CCM crystals in κ-carrageenan gels after exceeding 5–7 wt % of CCM.

### Thermal Properties

TGA thermograms of the CCM powder and PLA/PPC blends (including their analogues with CCM) are presented in Figure S6. In brief, the onset of degradation,^51^*T*_onset_, for CCM was evident above 207 °C (Table S1), indicating its stability during the fabrication process. This means that disadvantages typically associated with other bio-sourced indicators, such as anthocyanins, had been overcome.^13^ In this context, Chen et al.^52^ reported that the processing temperature of the CCM-containing food should not exceed 190 °C to avoid thermal degradation. The PLA/PPC blends thermally decomposed over the temperature range of 250–380 °C. Increasing the content of the PPC component resulted in *T*_onset_ shifting toward lower temperatures, with a subsequent broadening of the degradation peak. Similar findings were detected for the blends containing CCM, with minor differences in the *T*_onset_ and *T*_max_ values, since the CCM had been incorporated at a relatively low concentration. In summary, the TGA data suggest that the developed PLA/PPC indicators loaded with CCM can be successfully produced by melt extrusion/compression molding at the temperatures applied herein, without any significant thermal degradation, indicating the upscale potentiality of the material.

DSC analysis was carried out to further understand the effect of the CCM on the thermal behavior of the PLA/PPC blends. Figure S7 shows heating scans for the samples after erasing their thermal history; the numerical results are given in Table S2. The co-existence of PPC was reflected in heightened *T*_g_ at around 35 °C and lower Δ*H*_cc_ values for the binary blends. Likewise, adding the CCM further decreased the capacity for cold crystallization, suggesting hindered segmental mobility of the PLA chains.^24^ As a result, the presented blends and their analogues with CCM possessed a minimal degree of crystallinity, χ_C_, (below 1.5%), which predetermined their high level of transparency. Details on the thermal properties of the biodegradable films are provided in the Electronic Supporting Information (ESI).

### Rheological Properties

Oscillatory rheology is a sensitive method for studying change in polymer melt at a molecular level.^53^ Since polymeric materials are viscoelastic in nature, their mathematical description necessitates a complex variable approach. Cole–Cole representation of η* simplifies the interpretation of the complex plane diagram by employing a few fitting parameters.^36^Figure S8 contains Cole–Cole plots of η* for samples fitted with the model (eq 2). The findings revealed that the Cole–Cole model correlated reasonably well with the data when using the numerical parameters summarized in Table 1. All of the dependences showed an ideal semicircular form, evidencing a good compatibility^54^ of the PLA/PPC blends, since the deformation process was governed by a single relaxation time, λ_0_. Raising the PPC content and adding the CCM slightly diminished λ_0_ by decreasing the elasticity-to-viscosity ratio, hence intensifying the mobility of the polymer chains.^53,55^ This feature is contrary to heterogeneous polymeric systems,^55^ composites containing agglomerating fillers^56^ or systems that form three-dimensional (3D) polymeric networks.^57^ Moreover, the parameter *h* increased with the content of PPC, reflecting the broadening distribution of its *M*_w_.^36^ More importantly, the parameter η_0_, which was obtained by extrapolating the curve of the semicircle to the real axis, decreased by 34.2 and 45.4% after co-blending the PPC at the corresponding amounts. Small additions of the CCM further decreased the η_0_ viscosity, by 5.0, 8.6, and 12.4%, compared to the reference samples (PLA and two PLA/PPC blends). Thus, it can be stated that the addition of PPC improved fluidity while CCM molecules exerted a plasticizing effect; both factors consequently facilitated the processing of the molten-state mixtures.

**Table 1:** Numerical Results for the Parameters of the Cole–Cole Model

sample ID	η_0_ (Pa·s)	λ_0_ (ms)	*h* (−)
PLA/PPC 100/0	2994	24.1	0.334
PLA/PPC 100/0 + CCM	2844	22.8	0.325
PLA/PPC 80/20	1970	22.4	0.377
PLA/PPC 80/20 + CCM	1801	21.1	0.376
PLA/PPC 60/40	1634	22.9	0.393
PLA/PPC 60/40 + CCM	1413	20.1	0.379

### Mechanical Properties

**Figure 4:**
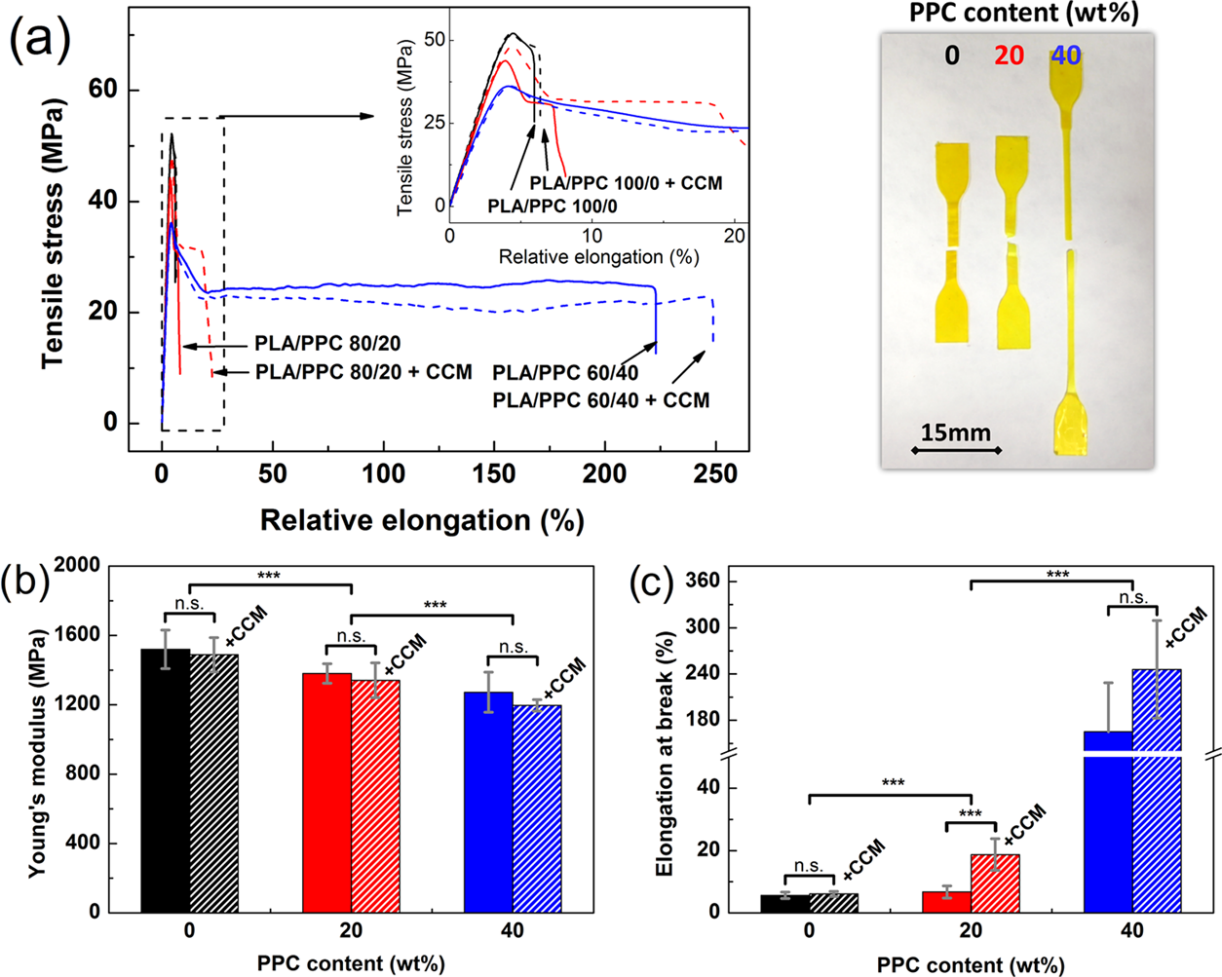
(a) Tensile curves and (b, c) mechanical parameters for the PLA/PPC blends (solid lines/columns) and their CCM-loaded analogues (dashed lines/columns); *** indicates a significant difference in comparison with variable values at *p* < 0.05. The digital image shows a macroscopic view of the samples after tensile testing to the point of failure.

Assessing the mechanical properties of biopolymers is crucial for packaging applications.^23,58^ The stress–strain curves and mechanical parameters for the PLA/PPC blends and their CCM-loaded analogues are presented in Figure 4. The curve for neat PLA exhibited yielding with subsequent fracture without necking (Figure 4a, inset) at very low elongation at break (5.6%). The binary blends showed elongation at break that increased with the rise in PPC content; only slight improvement was observed in blends containing 20 wt % of PPC, while samples with 40 wt % PPC experienced necking and improvement in elongation. This phenomenon is related to the existence of a certain threshold in PPC concentration above which a continuous phase arises in the binary blend. A dramatic change of this type, which does not follow the standard additive rule, can be attributed to potent interfacial adhesion between PLA and PPC.^47^ Additional improvements in such interfacial adhesion are achievable through modifiers, e.g., maleic anhydride^28^ or dicumyl peroxide.^48^ Change in composition was concurrently reflected in a gradual, albeit relatively small (less than 16%), decrease in Young’s modulus. For the corresponding blends, this decrease was slightly lower than findings in pioneering work by Ma et al.,^47^ potentially attributable to different grades (molecular weight, branching, variance in stereoisomeric proportion) of the PLA and PPC components, or other factors related to the fabrication process. Considering the ANOVA, the addition of the PPC had statistically significant effects on Young’s modulus and elongation at break for the neat blends, as well as their CCM-loaded analogues (Figure 4b,c).

Attention was also paid to investigating the effect of CCM on the mechanical properties of the blends. Incorporating it notably increased elongation at break, explained by a decrease in intramolecular cohesion and greater free volume as a consequence.^17^ Free volume controls the molecular mobility of polymer segments, which correlates herein with the λ_0_ parameter extracted from the Cole–Cole model (Table 1). Despite being uniformly dispersed, the CCM slightly decreased Young’s modulus by acting as a plasticizer; this change was not statistically significant at a given significance level due to the large variance of the response data (Figure 4b,c).^17^ Similar behavior was observed by Marković et al.^21^ after embedding CCM into polyurethane. A contrary finding of enhanced Young’s modulus was observed by Liu et al.^14^ for κ-carrageenan films after introducing 1–3 wt % of CCM. The enhancements in their composites, though, stemmed from hydrogen bonding interactions between the CCM and κ-carrageenan matrix. To conclude, the mechanical properties of the proposed PLA/PPC indicators can be effectively tuned by simply varying the ratio of the constituent materials.

### Optical Properties

**Figure 5:**
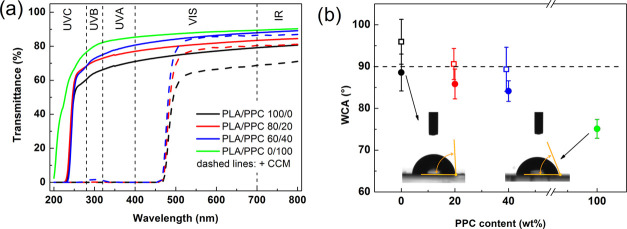
(a) UV–vis spectra and (b) WCA data for the PLA/PPC blends (solid lines/symbols) and their CCM-loaded analogues (dashed lines/open symbols).

The optical properties of packaging materials are important from both a functional and aesthetic point of view. It is necessary to prevent the photo-oxidative action of UV light, yet still provide high transparency for a clear view of the enclosed foodstuff.^20^Figure 5a presents transmittance spectra for the PLA/PPC films prior to and following the addition of CCM. The neat samples were highly transparent, transmitting ca 79–87% of light (*T*_700_) in the visible spectrum. Light transmittance generally increased in line with the increase in PPC content, indicating the good miscibility and compatibility of the polymer blends.^59^ These blends were also transparent to UVA and UVB radiation wavelengths, which are typical causes of photo-oxidation and photo-aging.^60^ The CCM-loaded analogues exhibited slightly lower, yet still reasonable transparency (up to 68–84%) in visible light (*T*_700_). Notably, the presence of the CCM dramatically decreased the transmission of unwanted UVA–UVC wavelengths, which is a highly desirable quality in food packaging. This behavior can be attributed to the remarkable capacity for UV absorption of the phenolic groups in the CCM molecules.^17^ The findings of this study affirm that the presence of CCM effectively improved the UV barrier properties of the PLA/PPC blends, while continuing to maintain high transparency in the visible spectrum. This marks out the developed material as highly suitable for storing food products and photosensitive substances.

### Interaction with Water

Figure 5b presents the details of the WCA of the samples. The WCA of neat PLA was 88.6 ± 4.4°, in general agreement with previous works.^61^ The binary blends exhibited a gradual decrease in WCA with PPC content, while neat PPC was found to have a WCA of 75.1 ± 2.2°. The relatively high variability of data represented by the error bars was attributed to possible nonuniformities in the surface roughness.^62^ CCM loading contributed to higher values for WCA, forming slightly hydrophobic films due to the hydrophobic nature of the phenyl rings in the CCM molecules.^16,20^ Although greater WCA often correlates with the decrease in polymer chain distance,^22^ we believe that in this study the CCM increased the average distance between the PLA/PPC chains, corresponding to reduced η_0_ viscosity and Young’s modulus in the CCM-loaded formulations (Figures S8 and 4b). The presence of nonpolar molecular segments in the CCM molecules, therefore, represented a dominating contribution in the increase of WCA.

### Analysis of Water Vapor Permeability

**Figure 6:**
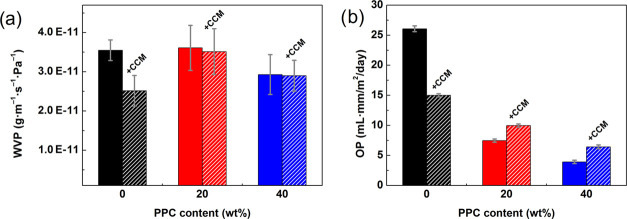
(a) Water vapor permeability (WVP) and (b) oxygen permeability (OP) of the PLA/PPC blends (solid columns) and their CCM-loaded analogues (dashed columns).

Recent research^22^ has shown that the presence of CCM slightly improved the water vapor barrier properties of LDPE films, as a result of intramolecular interactions decreasing the distance between CCM molecules and LDPE chains. Figure 6**a** reveals that the neat PLA that served as a reference exhibited a WVP value of around 3.5 × 10^–11^ g m^–1^ s^–1^ Pa^–1^. This value decreased after incorporating the CCM, which can be attributed to the aforementioned interactions between the PLA and CCM. In comparison, the PLA/PPC blends exhibited similar WVP values but the enhancing effect of the CCM was less pronounced, most likely due to the occurrence of weaker interactions between the CCM and binary matrix, as evidenced by the FTIR analysis (Figure 2). The results indicate that films with suggested compositions exhibited an adequate capacity for water vapor resistance comparable to neat PLA, a material widely utilized in packaging.^2^

### Oxygen Permeability Analysis

An important aim is to develop a film with high oxygen barrier functionality to restrict the oxidation of food. To this end, oxygen permeability (OP) was calculated from the oxygen transmission rate (OTR) for all of the prepared films and their CCM-loaded counterparts; the results are presented in Figure 6b. It is known from the literature^35^ that PPC is usually characterized by notably lower OP values than neat PLA. This is why co-blending PPC with PLA appeared to be an effective strategy for enhancing the oxygen barrier performance of the PLA-based films. While neat PLA attained an OP value of 26.0 ± 0.4 mL·mm/m^2^/day, lower ones of 7.6 ± 0.2 and 4.0 ± 0.1 mL·mm/m^2^/day were observed for the binary blends with PPC at 20 and 40 wt %, respectively. Such enhancement stemmed from the impermeability of the PPC, as its polymer chains could have formed more tortuous pathways that impeded the permeation of oxygen molecules through the blends. After incorporating the CCM, the OP of the PLA film decreased to 15.0 ± 0.2 mL·mm/m^2^/day, most probably caused by the heightened χ_C_ of the PLA/CCM film, as discerned by DSC (Table S2). In this context, it is possible that the crystallization phenomena complicated the movement of oxygen molecules, inevitably reducing the OP of the PLA/CCM film. On the contrary, the presence of the CCM slightly increased the OP values for the PLA/PPC binary blends. This most likely occurred through the loosening of the polymer structure, despite the homogeneous dispersion of all constituent parts (Figure 3), although the extent of such increase was rather marginal. The OP analysis revealed that the PLA/PPC blends, even after being supplemented with CCM, possessed significantly better oxygen barrier properties than widely used PLA.

### Release Kinetics

**Figure 7:**
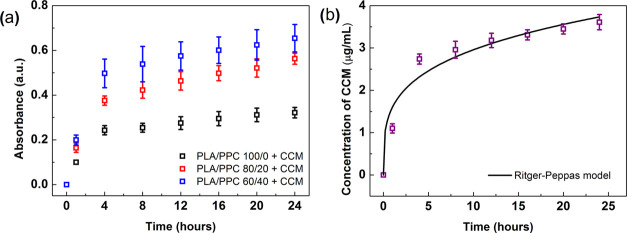
(a) Release kinetics for CCM-loaded PLA/PPC films, followed by measuring the absorbance of the solution at 428 nm, the characteristic absorption peak for CCM. The kinetics are presented for the three films with different PPC concentrations. (b) Concentration of released CCM in ethanol over time, for the representative sample (PLA/PPC 60/40).

The release kinetics of an active compound is a crucial aspect when developing smart packaging materials. Figure 7a shows the release kinetics for the CCM released from the PLA/PPC films in ethanol, a common fatty food simulant. The kinetics were followed by measuring the absorbance of the solution at 428 nm, the characteristic absorption peak for CCM. To compare the release behavior for individual samples, the data was modeled according to the Ritger–Peppas equation^63^ for nonswellable polymeric devices
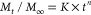
10where *M*_t_ and *M*_∞_ correspond to the cumulative amounts of CCM released at time *t* and infinite time, *K* is a material constant related to the characteristics of the macromolecular network system and active component, and *n* is the diffusional exponent. The short-time approximation was made for release that 60% of the CCM would be extracted (i.e., a fractional release, *M*_t_/*M*_∞_ ≤ 0.60); the relevant numerical parameters are summarized in Table S3. The amount of CCM released into the solution increased with PPC concentration because of the greater affinity of the indicators for polar solutions, thereby facilitating penetration of the solvent molecules through the structure of the blend. This behavior also reflected the absolute ability of the PLA/PPC films to release CCM, as given by the *M*_∞_ values. The rate of release denoted by the *K* constant also increased from 0.211 to 0.263 and 0.326 h^–1^ after co-blending the corresponding amounts of PPC. The sample with the best release capability was able to release up to 3.61 μg/mL of CCM within 24 h; see Figure 7b. These results indicate that the developed films could increase the shelf life of fatty foodstuffs by inhibiting oxidative stress over time.^22,64^

### Antioxidant Activity

**Figure 8:**
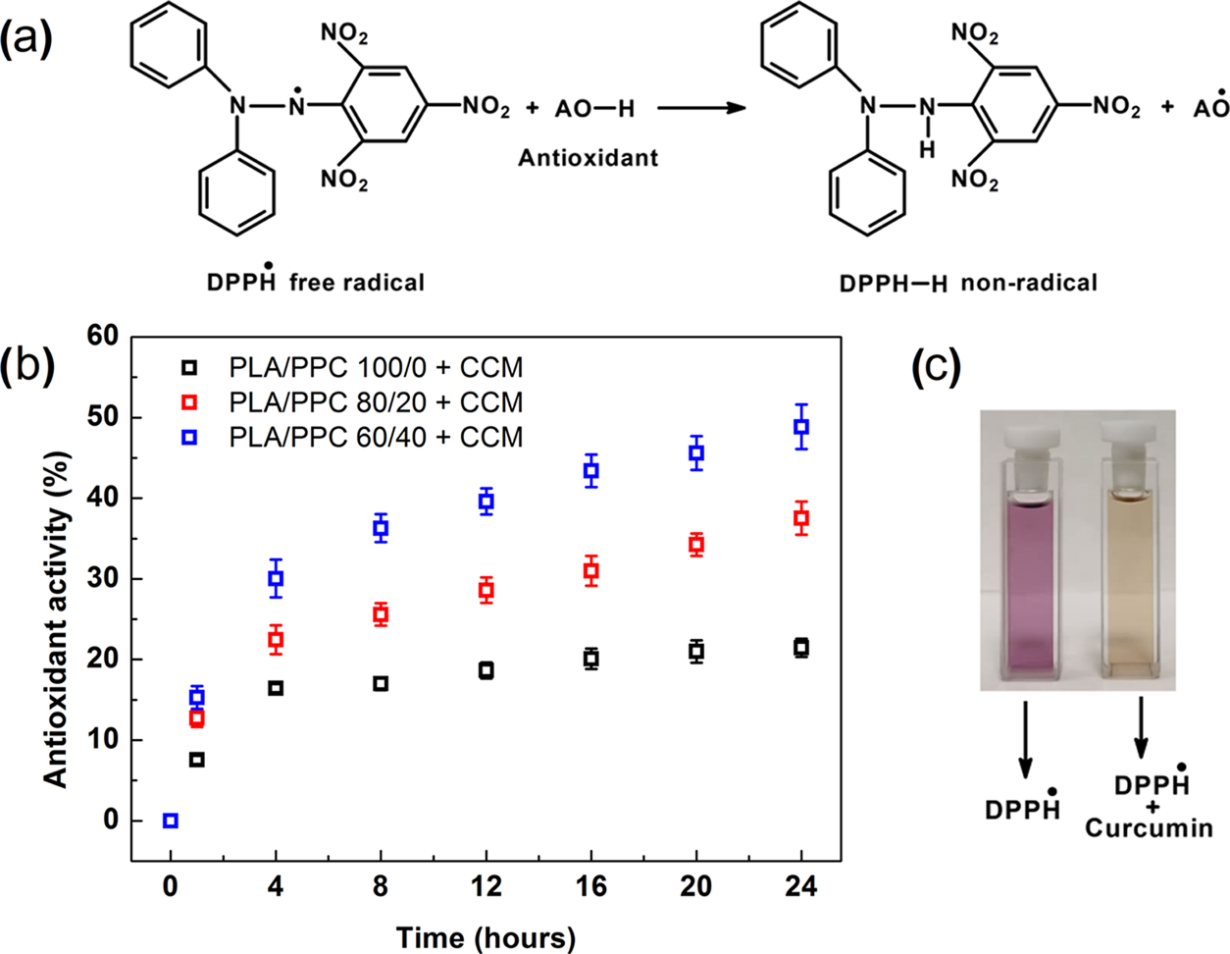
(a) Reaction mechanism of the DPPH^•^ with the natural antioxidant. (b) Antioxidant activity of CCM-loaded PLA/PPC films as a function of time. (c) Macroscopic picture showing the scavenging activity of the CCM released in ethanol.

A standard DPPH^•^ assay was employed as a suitable method^22,65^ to evaluate the antioxidant potential of the CCM-loaded PLA/PPC films in terms of lipid oxidation.^66^ It should be emphasized that the antioxidant testing was performed using 2 mL extract; therefore, the absolute amount of the CCM released during the test was 2× higher, compared to the data in Figure 7b. As shown in Figure 8, antioxidant activity increased with the increase in PPC content and time, due to the release of CCM into the ethanol solution. The greater amount of CCM molecules in the solution provided more hydrogen radicals, which subsequently stabilized a higher number of DPPH^•^ free radicals that converted into their stabilized form (DPPH–H). This process was observed macroscopically, evidencing a significant change in color by the DPPH^•^ solution from violet to yellow; the exact extent of change in hue depended on the antioxidant capability of the PLA/PPC films (Figure 8, inset).^22,67^ Out of the samples tested, the film with the greatest antioxidant capability was based on the PLA/PPC 60/40 mixture. This film exhibited increased antioxidant activity (during 24 h) by a factor of 2.3× compared to the reference (49.3 ± 2.7 vs 21.2 ± 1.1%). The antioxidant ability of this film even exceeded that of a synthetic-based LDPE counterpart with CCM at 5 wt %, which demonstrated an antioxidant activity of 44.5% after 24 h.^22^ According to the literature,^68^ the ethanol solution represents the food systems with a higher content of fat, such as meat. Thus, the antioxidant activity of presented CCM-loaded PLA/PPC packaging films can be correlated with the reduction of lipid oxidation, and consequent extension of the shelf life of fatty foodstuffs.^65,69^

### Overall Migration Analysis

Overall migration (OM) tests were carried out to simulate the migration effects of a foodstuff according to current legislation (EU Commission Regulation No. 10/2011). Figure S9 shows that the transfer of substances to Tenax (a dry food simulant) increased in parallel with an increase in PPC content, while the additional increment was caused by the presence of the CCM. This behavior was attributed to the adhesive qualities of the PPC, which is an amorphous polymer with a low *T*_g_^PPC^ of around 35 °C (Table S2). Despite the heightened migration levels, OM values remained within the accepted range, i.e., below 10 mg/dm^2^. The collected data proved compliance with migration limits; thus, the PLA/PPC/CCM films should not endanger human health or deteriorate organoleptic properties.

### Detection of Ammonia Vapors

**Figure 9:**
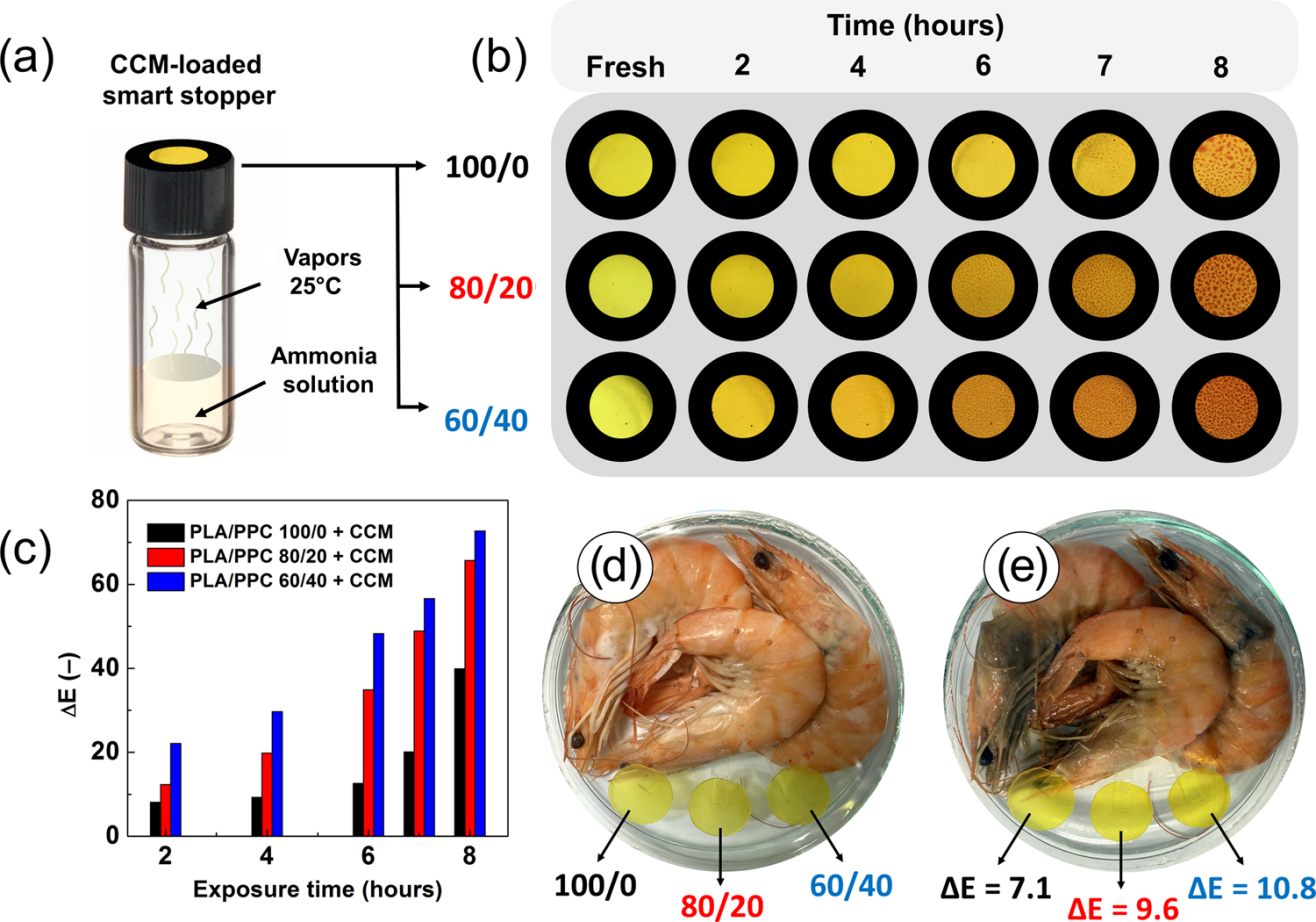
(a) Schematic illustration of the testing assembly. (b) Digital photographs and (c) the corresponding color changes expressed as Δ*E* values of CCM-loaded PLA/PPC indicators at different times of exposure to NH_3_ vapors. Shrimps sealed in a Petri dish at the beginning of the test (d) and on day 5 (e).

The onset of bacterial proteolysis is considered the primary biochemical indicator of food spoilage. The presence of free amino acids and nitrogen compounds, such as NH_3_, is associated with the nauseating odors of rotting food.^70^ To examine the NH_3_ sensing capability of the PLA/PPC-based indicators, an aqueous ammonia solution was applied, from which NH_3_ is easily volatilized.^17^Figure 9a,b displays the testing assembly and the corresponding color changes of the PLA/PPC indicator films upon exposure to NH_3_ vapors over time. As can be seen, CCM-loaded films started to change the color after 2 h of exposure to NH_3_ vapors; the color change was proved by the CIELab color analysis that resulted in Δ*E* > 5 in all of the cases (Figure 9c), indicating that the color change was perceivable by the naked eye.^46^ The intensity of the color change from yellow to orange/red was increasing with the exposure time to NH_3_ vapors, which was accompanied by a significant increase in the Δ*E* values, from 8.1 to >39.9 (within the studied time period from 2 to 8 h) for the PLA film supplemented with the CCM. The NH_3_ sensing mechanism can be explained as follows: water molecules adhere to the CCM-loaded indicator films, where they react with NH_3_ vapor to form NH_4_^+^ and OH^–^ ions. The hydroxyl ions react with the phenolic hydroxyl group of the CCM, forming a phenolic oxygen anion. Such electron redistribution is manifested through modified optical properties.^17^ Importantly, Figure 9c further shows that Δ*E* values at specific time intervals increased with the PPC concentration in the polymer blend, indicating an improved NH_3_ vapor sensitivity. Based on the CIELab analysis, the PLA/PPC 60/40 indicator film showed the best responsiveness that produced the most significant color change, in terms of Δ*E* values, among the other samples (the change from 22.1 to >72.7, within the time period of 2–8 h). This effect was attributed to the higher hydrophilicity of the PPC-containing blends (Figure 5b), which promotes the adhesion of water molecules and accelerates acid–base reactions. Based on the results obtained, the CCM-loaded PLA/PPC indicators are considered effective at detecting NH_3_, they provide color changes that can be easily distinguished by the naked eye, and it is possible to tune their sensitivity by merely altering the ratio of the given polymers. Apart from their great potentiality as indicators on smart packaging for foodstuffs, they are also applicable as on-demand vapor sensors for detecting NH_3_ gas in industrial and environmental applications.

### Food Spoilage Test

The microbiological spoilage of most animal-based proteins relates to the production of volatile basic nitrogen in the form of ammonia, dimethylammonium, and trimethylamine. These compounds can affect the pH value of the environment, which in turn may trigger a change in the color of CCM.^14^ Therefore, the CCM-loaded PLA/PPC indicators were adopted as colorimetric indicators of the spoilage process (Figure 9d,e). As is evident, the indicators changed color from yellow to light orange within 5 days, indicating the formation of volatile amines during the spoilage of shrimps. In all of the cases, the CIELab analysis resulted in Δ*E* > 5, indicating visually perceivable color changes; however, the PPC-containing indicator (60/40) demonstrated markedly higher sensitivity (Δ*E* up to 10.8). To conclude, the developed PLA/PPC/CCM indicators are capable of signaling the process of microbial spoilage and show great promise as an alternative material for smart packaging applications. Despite this, future optimizations related to their sensitivity are necessary prior to widespread adoption by the food industry.

## Conclusions

In conclusion, we have developed biodegradable and mostly bio-based films that can be used as both, sustainable packaging and smart colorimetric indicators, composed of PLA, PPC, and CCM suitable for smart food packaging applications. Homogeneous PLA blends with PPC at the content of up to 40 wt % and a constant CCM loading of 2 wt % were produced by industrially viable techniques, such as melt extrusion and compression molding, without any signs of degradation. The mechanical, physical, and chemical properties of the films were controlled by the PLA/PPC ratio and by the addition of CCM. Increasing the amount of PPC resulted in a notably lower viscosity, facilitating the processing of the material. Despite a slightly lower Young’s modulus (less than 16%), the samples showed exceptionally high elongation at break (a 43-fold increase with respect to PLA). The CCM also efficiently blocked UV light yet remarkable transparency was maintained, exhibiting *T*_700_ of about 68–84%. The CCM-loaded blends showed a slightly higher hydrophobicity than their neat analogues, similar WVP values to widespread PLA (3.5 × 10^–11^ g m^–1^ s^–1^ Pa^–1^), and even lower OP values, in spite of a plasticizing effect caused by the CCM. The loosened structure of the PLA/PPC blends benefited the release of the active compound to the surrounding environment, providing potent antioxidant activity (up to 49.3 ± 2.7% within 24 h) in terms of lipid oxidation. Migration levels were tested using a dry food simulant and found to be below EU Commission limits, marking the PLA/PPC blends out as a safe food contact material. Furthermore, the blends exhibited macroscopical color changes with a tunable intensity response for NH_3_ vapor, which could be adopted for on-site detection of the freshness of foodstuffs. Owing to the combined qualities of UV protection, optical transparency, barrier performance, antioxidant activity, NH_3_ sensitivity, and the ability to monitor food freshness, the developed bio-formulations represent a promising breakthrough in the technology of smart packaging and associated applications.

## Supporting Information Available

Linear fit for the determination of CCM concentration; FTIR spectrum for neat PPC; SEM micrographs of the neat PLA/PPC matrices; SEM micrograph of CCM crystals in the powder form; XRD patterns of the investigated blends; detailed description of the TGA and DSC data (including numerical data); Cole–Cole plots for the investigated blends; diffusional parameters for CCM release; and overall food migration using Tenax (graphical data) (PDF)
