# Supplementary material for: Biodegradable Films of PLA/PPC and Curcumin as Packaging Materials and Smart Indicators of Food Spoilage
Source: ACS Appl Mater Interfaces. 2022 Mar 18;14(12):14654–67. doi: 10.1021/acsami.2c02181 (PMC8972250; doi:10.1021/acsami.2c02181)
Supplement: Supplementary file 1 — am2c02181_si_001.pdf [file am2c02181_si_001.pdf]

Supporting Information

Biodegradable Films of PLA/PPC and Curcumin as Packaging Materials and Smart Indicators of Food Spoilage

Martin Cvek[a], Uttam C. Paul[b], Jasim Zia[b], Giorgio Mancini[b], Vladimir Sedlarik[a], Athanassia Athanassiou[b,]*

[a] Centre of Polymer Systems, University Institute, Tomas Bata University in Zlin, Trida T. Bati 5678, 760 01 Zlin, Czech Republic

[b] Smart Materials, Istituto Italiano di Tecnologia, Via Morego 30, 161 63 Genoa, Italy

KEYWORDS

Polylactic acid; polypropylene carbonate; curcumin; smart food packaging; chemical sensor; bioplastics; indicator.

*Corresponding Author: athanassia.athanassiou@iit.it

Contents

Figure S1: Linear fit for the determination of CCM concentration. S3

Figure S2: FTIR spectrum for neat PPC with highlighted characteristic wavenumbers, constituting the reference for the PLA/PPC blends. S3

Figure S3: SEM micrographs and magnified images of the neat PLA/PPC matrices. S4

Figure S4: SEM micrograph of CCM crystals in the powder form S4

Figure S5: XRD patterns for the PLA/PPC blends (*solid lines*) and their CCM-loaded analogues (*dashed lines*). Diffraction peaks for the powder CCM are indexed for the identification purpose. S4

Figure S6: (*a*) TGA and (*b*) DTG spectra for pure CCM powder, the PLA/PPC blends (*solid lines*) and their CCM-loaded analogues (*dashed lines*). S6

Figure S7: Normalized DSC curves for the PLA/PPC blends (*solid lines*) and their CCM-loaded analogues (*dashed lines*) corresponding to the second heating cycle. S7

Figure S8: Cole-Cole plots for the PLA/PPC blends (*solid symbols*) and their CCM-loaded analogues (*open symbols*); the best predictions of the Cole-Cole model are represented by the solid and dashed lines, respectively. S8

Figure S9: Overall food migration analysis using Tenax® on the PLA/PPC blends (*solid columns*) and their CCM-loaded analogues (*dashed columns*). S9

Table S1: Characteristic temperatures for thermal decomposition as determined by TGA. S6

Table S2: DSC data for the PLA/PPC blends and their CCM-loaded analogues gathered after erasing the thermal history. S8

Table S3: Diffusional parameters for diffusional CCM release from the PLA/PPC films. S8

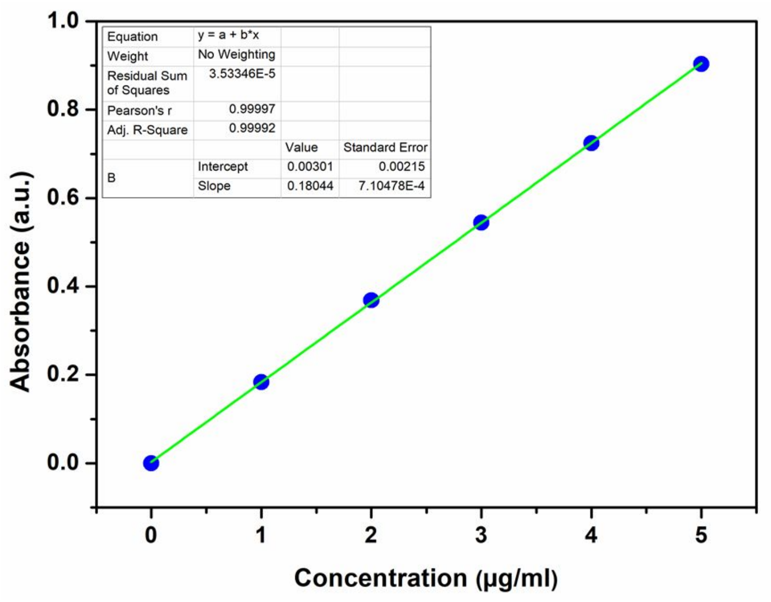

Figure S1: Linear fit for the determination of CCM concentration.

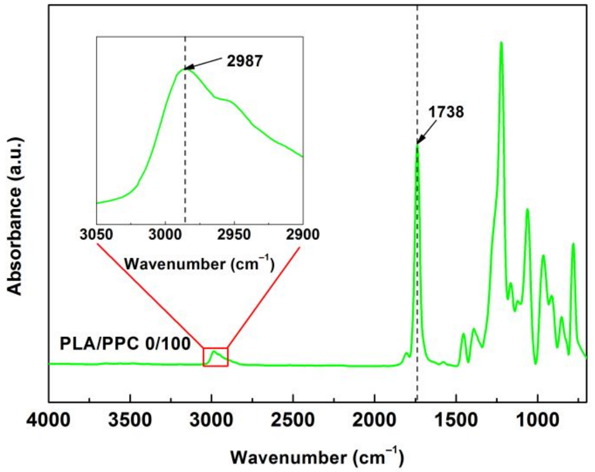

Figure S2: FTIR spectrum for neat PPC with highlighted characteristic wavenumbers, constituting the reference for the PLA/PPC blends.

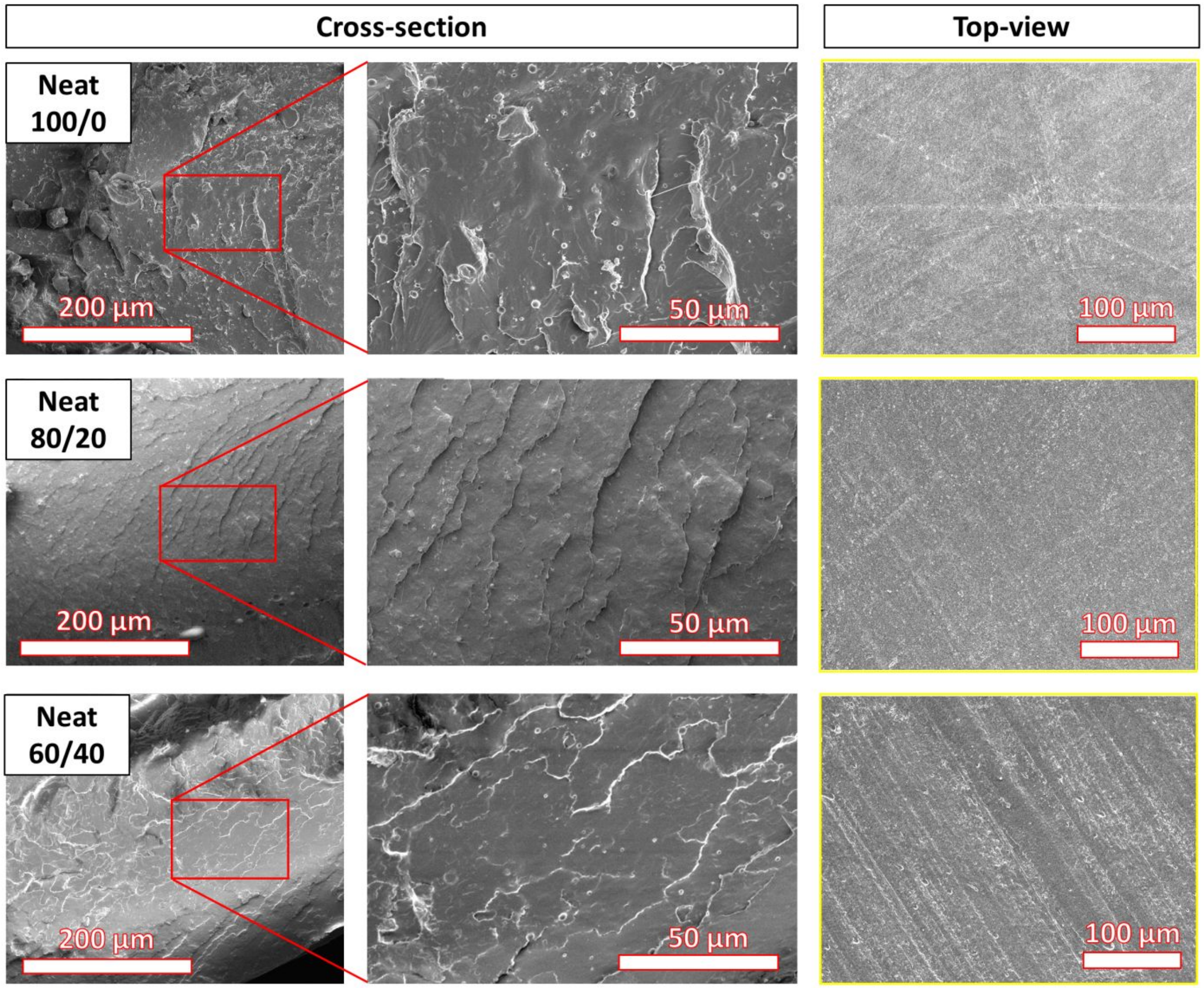

Figure S3: SEM micrographs and magnified images of the neat PLA/PPC matrices.

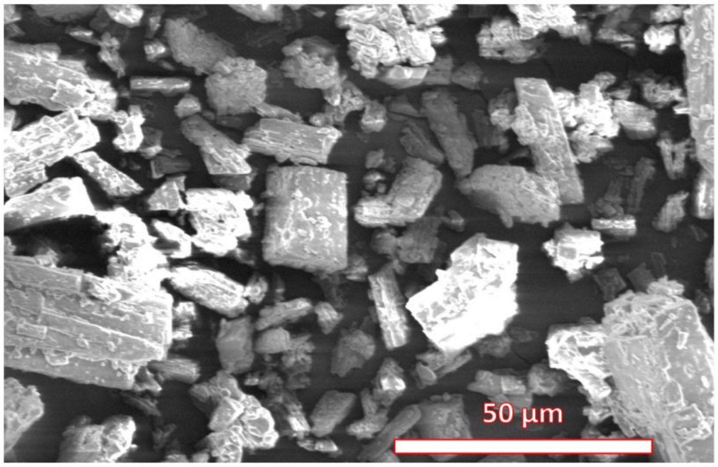

Figure S4: SEM micrograph of CCM crystals in the powder form.

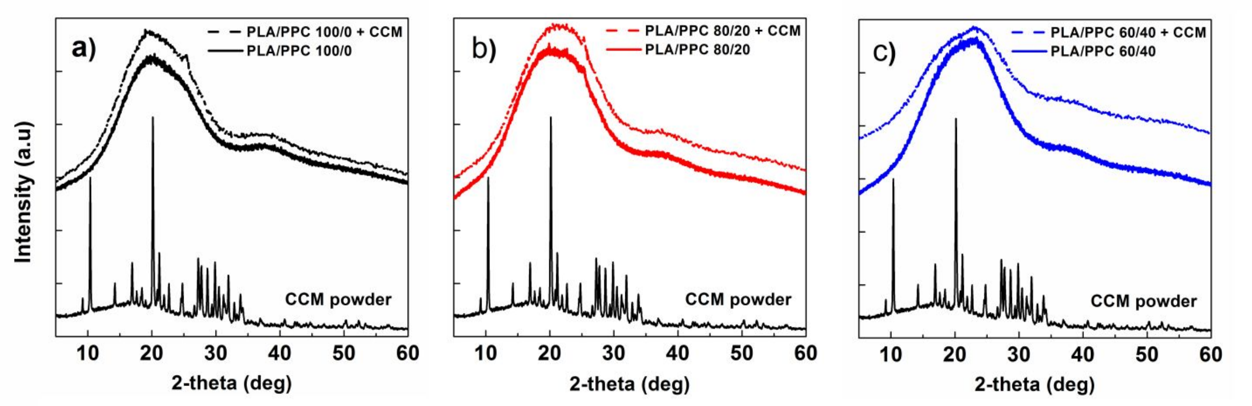

Figure S5: XRD patterns for the PLA/PPC blends (*solid lines*) and their CCM-loaded analogues (*dashed lines*). Diffraction peaks for the powder CCM are indexed for the identification purpose.

Thermal properties. The investigation was made to gauge the thermal stability of neat CCM, which served as an active element in fabrication of the sensors (**Figure S6a**). Note that the onset of thermal degradation, T_{onset}, was defined as the temperature at which 2 wt% of total mass was volatilized.[1] DTG formalism (**Figure S6b**) was applied to aid the interpretation of CCM decomposition, which was revealed to be a slow, dual-stage process. Starting from T_{onset}, the first stage ended at approximately the local minimum of the DTG curve (411°C), which involved a peak (T_{max}) at 334°C. The latter stage proceeded and exhibited an end point at around 582°C, until the almost complete decomposition (1.0 wt% of residuals). This dual-stage mechanism stems from the chemical structure of the CCM molecule, which shows the preferential decomposition of substituent groups followed by the benzene rings.[2]

Neat PLA demonstrated no sign of having absorbed water, a T_{onset} value of 308°C and continuous weight loss was observed until 381°C, with T_{max} occurring at 359°C. Unlike CCM, PLA exhibited a single-stage process of thermal degradation, due to the occurrence of just one dominating mechanism (intramolecular transesterification) alongside other non-radical and radical reactions.[1, 3] After co-blending it with PPC, T_{onset} shifted towards lower temperatures (263°C and 247°C), as did T_{max} (355°C and 303°C), in parallel with increase in the content of PPC from 20 to 40 wt%, since PPC has lower temperature stability than PLA.[4] Moreover, the DTG spectra revealed the broadening and overlapping of peaks, which evidenced the co-existence of both components in the binary mixtures.

Thermograms of the CCM-loaded samples generally mimicked the features of neat PLA and corresponding PLA/PPC blends as a consequence of the low concentration of load. The presence of CCM slightly deteriorated the thermal stability of neat PLA, reflected in a negligible difference in T_{onset} and slight drop (5°C) in T_{max} value. Conversely, the CCM improved the thermal stability of both PLA/PPC blends as evidenced by a shift in points of inflexion. The studied blends exhibited a rise in T_{onset} by 4°C and 10°C, while T_{max} values increased by 2°C and 9°C, respectively, compared to samples without CCM. Following combustion (above 600°C), all the samples yielded a char residue of less than 2 wt%. This indicates that thermal degradation reactions mostly proceeded by formation of CO and CO_2 volatile gases.[1] The numerical data set of TGA results is given in Table S1.

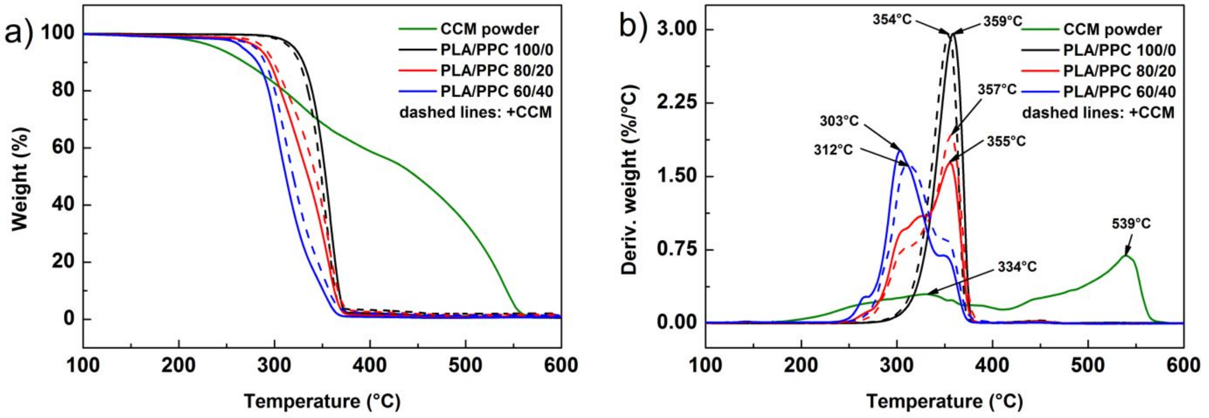

Figure S6: (*a*) TGA and (*b*) DTG spectra for pure CCM powder, the PLA/PPC blends (*solid lines*) and their CCM-loaded analogues (*dashed lines*).

Table S1: Characteristic temperatures for thermal decomposition as determined by TGA.

Sample ID	T_{onset} (°C)	$T_{5\%}$ (°C)	$T_{50\%}$ (°C)	$T_{95\%}$ (°C)	$\Delta T_{5–95}$ (°C)
CCM	207	240	449	551	311
PLA/PPC 100/0	308	321	353	371	50
PLA/PPC 100/0 + CCM	304	317	349	367	50
PLA/PPC 80/20	263	283	335	367	84
PLA/PPC 80/20 + CCM	273	289	343	372	83
PLA/PPC 60/40	247	267	312	359	92
PLA/PPC 60/40 + CCM	252	280	319	365	85

Figure S7 and Table S2 present DSC data related to the PLA/PPC blends and their CCM-loaded analogues. It should be emphasized that the evident exothermic peaks, associated with cold crystallization, related to semi-crystalline PLA due to the amorphous character of the PPC.[5] Neat PLA exhibited a typical DSC trace, comprising a T_g^{PLA} phase transition at around 59°C, along with crystallization and melting peaks at around 122°C and 149°C, respectively. Its ΔH_{cc} and ΔH_m exhibited similar values, confirming that crystallization of the PLA only occurred during the heating phase.[6] In the binary blends, the co-existence of PPC was reflected in the additional T_g^{PPC} at around 35°C and reduced ΔH_{cc} values, without any significant effect on T_{cc} values. Other researchers[7] have observed that co-blending PPC with PLA decreases values for T_{cc}, which can be attributed to the decomposition of the PPC into low-molecular segments. In our case, the data suggest that thermal degradation during the PLA/PPC processing stage was limited. Inclusions of CCM crystals further decreased the capacity for cold crystallization, suggesting the segmental mobility of the PLA chains was hindered.[8] As a result, the PLA molecular chains in the CCM-loaded samples could not gain sufficient mobility to undergo rearrangement into orderly crystals, a phenomenon accompanied by fluctuation in values for ΔH_{cc} and, consequently, ΔH_m. The opposite was discerned in a study on PLA loaded with cellulose nanocrystals, which served as nucleating agents for promoting crystallization.[8] The total χ_C for PLA is generally dependent on the fabrication process involved; in this context, Carrasco et al.[3] reported 4.1–4.5% for injected material and 7.8–8.4% for extruded/injected material, respectively; later, Flodberg et al.[5] reported an χ_C of 1.2% for cast PLA films. Herein, after calculating the value for total χ_C (Equation 1), it became obvious that the PLA/PPC blends possessed minimal crystallinity (an χ_C of less than 1.5%), and neither the presence of PPC nor CCM had any apparent effect on crystallinity.[7] This phenomenon is a predisposition for the blends possessing good optical properties applicable in packaging applications.

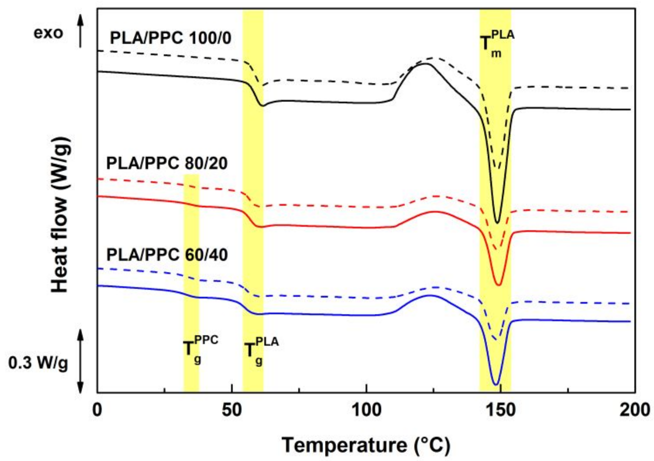

Figure S7: Normalized DSC curves for the PLA/PPC blends (*solid lines*) and their CCM-loaded analogues (*dashed lines*) corresponding to the second heating cycle.

Table S2: DSC data for the PLA/PPC blends and their CCM-loaded analogues gathered after erasing the thermal history.

Sample ID	T_g^{PLA}(°C)	T_g^{PPC}(°C)	T_{cc} (°C)	ΔH_{cc} (J/g)	T_m (°C)	ΔH_m (J/g)	χ_C (%)
PLA/PPC 100/0	59.5	N/D	122.4	20.3	148.8	20.7	0.5
PLA/PPC 80/20	55.7	34.7	126.2	9.0	149.3	9.8	1.1
PLA/PPC 60/40	54.6	32.7	124.1	11.3	148.2	12.0	1.3
PLA/PPC 100/0 + CCM	59.2	N/D	125.5	13.5	148.8	14.5	1.1
PLA/PPC 80/20 + CCM	56.4	35.8	127.4	6.7	148.8	6.8	0.2
PLA/PPC 60/40 + CCM	56.0	34.8	126.3	6.5	148.5	6.9	0.8

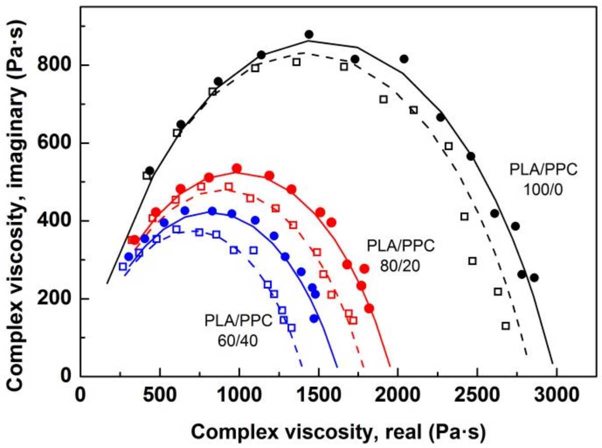

Figure S8: Cole-Cole plots for the PLA/PPC blends (*solid symbols*) and their CCM-loaded analogues (*open symbols*); the best predictions of the Cole-Cole model are represented by the solid and dashed lines, respectively.

Table S3: Diffusional parameters for diffusional CCM release from the PLA/PPC films.

Sample ID	M_∞ (μg/mL)	K (h^{-1})	n (–)	R^2
PLA/PPC 100/0 + CCM	3.59	0.211	0.277	0.983
PLA/PPC 80/20 + CCM	4.50	0.263	0.306	0.986
PLA/PPC 60/40 + CCM	4.92	0.326	0.266	0.978

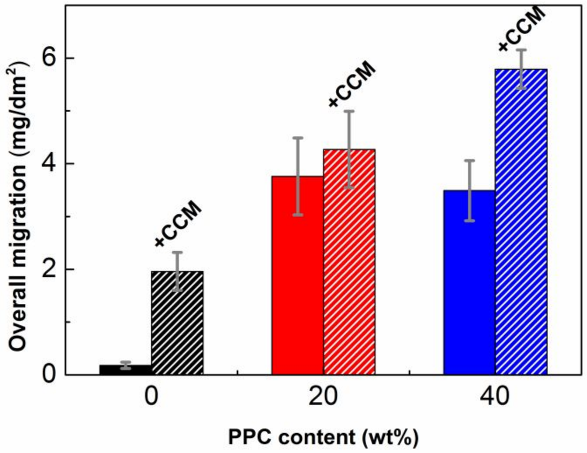

Figure S9: Overall food migration analysis using Tenax® on the PLA/PPC blends (*solid columns*) and their CCM-loaded analogues (*dashed columns*).

Acknowledgements
The author M.C. gratefully acknowledges the projects DKRVO (RP/CPS/2020/006) and (RP/CPS/2022/007) supported by the Ministry of Education, Youth and Sports of the Czech Republic. The authors acknowledge the Sustainability Initiative of the IIT. The authors also thank Ms. Lara Marini (Istituto Italiano di Tecnologia) for conducting the TGA and DSC measurements.

Declaration of interest: The authors declare no competing financial interests.

References

(1) Mngomezulu, M. E.; Luyt, A. S.; Chapple, S. A.; John, M. J. Poly(Lactic Acid)-Starch/Expandable Graphite (PLA-Starch/EG) Flame Retardant Composites. *J. Renewable Mater.* **2018,** *6*, 26–37.

(2) Chen, Z. P.; Xia, Y.; Liao, S.; Huang, Y. H.; Li, Y.; He, Y.; Tong, Z. F.; Li, B. Thermal Degradation Kinetics Study of Curcumin with Nonlinear Methods. *Food Chem.* **2014,** *155*, 81–86.

(3) Carrasco, F.; Pages, P.; Gamez-Perez, J.; Santana, O. O.; Maspoch, M. L. Processing of Poly(Lactic Acid): Characterization of Chemical Structure, Thermal Stability and Mechanical Properties. *Polym. Degrad. Stab.* **2010,** *95*, 116–125.

(4) Muthuraj, R.; Mekonnen, T. Carbon Dioxide-Derived Poly(Propylene Carbonate) as a Matrix for Composites and Nanocomposites: Performances and Applications. *Macromol. Mater. Eng.* **2018,** *303*, 1800366.

(5) Flodberg, G.; Helland, I.; Thomsson, L.; Fredriksen, S. B. Barrier Properties of Polypropylene Carbonate and Poly(Lactic Acid) Cast Films. *Eur. Polym. J.* **2015,** *63*, 217–226.

(6) Battegazzore, D.; Bocchini, S.; Frache, A. Crystallization Kinetics of Poly(Lactic Acid)-Talc Composites. *Express Polym. Lett.* **2011,** *5*, 849–858.

(7) Yao, M.; Deng, H.; Mai, F.; Wang, K.; Zhang, Q.; Chen, F.; Fu, Q. Modification of Poly(Lactic Acid)/Poly(Propylene Carbonate) Blends Through Melt Compounding with Maleic Anhydride. *Express Polym. Lett.* **2011,** *5*, 937–949.

(8) Mahmud, S.; Long, Y.; Abu Taher, M.; Xiong, Z.; Zhang, R. Y.; Zhu, J. Toughening Polylactide by Direct Blending of Cellulose Nanocrystals and Epoxidized Soybean Oil. *J. Appl. Polym. Sci.* **2019,** *136*, 48221.
